# Women’s empowerment through homestead food production in rural Bangladesh

**DOI:** 10.1186/s12889-022-12524-2

**Published:** 2022-01-19

**Authors:** Sarah Dupuis, Monique Hennink, Amanda S. Wendt, Jillian L. Waid, Md Abul Kalam, Sabine Gabrysch, Sheela S. Sinharoy

**Affiliations:** 1 Hubert Department of Global Health, Rollins School of Public Health, Mailstop 1518-002-7BB, Emory University, Atlanta, GA 30322 USA; 2 Research Department 2, Potsdam Institute for Climate Impact Research (PIK), Member of the Leibniz Association, P.O. Box 60 12 03, 14412 Potsdam, Germany; 3 Heidelberg Institute of Global Health, Heidelberg University, Im Neuenheimer Feld 324, 69120 Heidelberg, Germany; 4 Bangladesh Country Office, Helen Keller International, Rd No 82, Dhaka, 1212 Bangladesh; 5 Institute of Public Health, Charité – Universitätsmedizin Berlin, corporate member of Freie Universität Berlin and Humboldt-Universität zu Berlin, Charitéplatz 1, 10117 Berlin, Germany

**Keywords:** Agency, Empowerment, Gender, Agriculture, Nutrition-sensitive

## Abstract

**Background:**

Women in rural Bangladesh face multiple, inter-related challenges including food insecurity, malnutrition, and low levels of empowerment. We aimed to investigate the pathway towards empowerment experienced by women participating in a three-year nutrition-sensitive homestead food production (HFP) program, which was evaluated through the Food and Agricultural Approaches to Reducing Malnutrition (FAARM) cluster-randomized controlled trial.

**Methods:**

We conducted 44 in-depth interviews and 12 focus group discussions with men and women in both intervention and control communities of the FAARM study site in rural, north-eastern Bangladesh. Using a modified grounded theory approach to data collection and analysis, we developed a framework to explain the pathway towards empowerment among HFP program participants.

**Results:**

The analysis and resulting framework identified seven steps towards empowerment: 1) receiving training and materials; 2) establishing home gardens and rearing poultry; 3) experiencing initial success with food production; 4) generating social or financial resources; 5) expanding agency in household decision-making; 6) producing renewable resources (e.g. farm produce) and social resources; and 7) sustaining empowerment. The most meaningful improvements in empowerment occurred among participants who were able to produce food beyond what was needed for household consumption and were able to successfully leverage these surplus resources to gain higher bargaining power in their household. Additionally, women used negotiation skills with their husbands, fostered social support networks with other women, and developed increased self-efficacy and motivation. Meanwhile, the least empowered participants lacked support in critical areas, such as support from their spouses, social support networks, or sufficient space or time to produce enough food to meaningfully increase their contribution and therefore bargaining power within their household.

**Conclusions:**

This study developed a novel framework to describe a pathway to empowerment among female participants in an HFP intervention, as implemented in the FAARM trial. These results have implications for the design of future nutrition-sensitive agriculture interventions, which should prioritize opportunities to increase empowerment and mitigate the barriers identified in our study.

**Trial registration:**

FAARM is registered with ClinicalTrials.gov (NCT02505711).

## Background

Women in rural Bangladesh commonly face a number of inter-related barriers to empowerment, which are exacerbated by undernutrition and limited social resources [1–3]. Malnutrition is common, with 12% of ever-married women aged 15–49 years in rural Bangladesh underweight, defined as a body mass index less than 18.5 [1]. Additionally, in 2011, only 21% of Bangladeshi women aged 18 and over were categorized as empowered in agriculture based on the Women’s Empowerment in Agriculture Index (WEAI) [4]. Studies have observed positive associations between women’s empowerment and nutrition outcomes for both women and children in Bangladesh and South Asia more broadly, although the evidence is mixed [1, 5–7]. Homestead food production (HFP) programs aim to improve both nutrition and empowerment by training women to produce nutrient-rich foods close to their homes. These nutrition-sensitive interventions aim to build knowledge and skills primarily in vegetable gardening and poultry rearing and often incorporate other complementary trainings, such as the marketing of produce [8, 9]. Helen Keller International (HKI) began implementing HFP interventions in Bangladesh in the late 1980s to address micronutrient deficiency and improve the nutritional status of all household members, especially young children [10, 11]. Over time, HKI developed an enhanced HFP model, which includes an increased emphasis on women’s empowerment. The HFP model used in the FAARM trial, which this study examines, included group formation for agriculture training, skill building, and health and nutrition education; productive asset transfer; household visits and counseling to reinforce training messages; group leadership opportunities for selected participants and their families; and market linkages for income generation [9].

The pathways through which HFP programs may influence women’s empowerment have been under-studied [12]. Existing research relies heavily on quantitative measurement of empowerment as an outcome, including through the administration of the WEAI. Less research exists on the process or pathway to empowerment through HFP programs. Qualitative data on empowerment are limited and primarily used to complement the quantitative WEAI results [8]. Additionally, while the WEAI collects data from women and men, most existing qualitative research has only involved women. The emic perspectives of men are needed, as support from men and boys is a key resource for women’s empowerment [13].

To address these gaps, this study aimed to investigate the pathway towards increased empowerment that women experience in HFP programs. We used Kabeer’s definition of empowerment, “The ability to make strategic life choices in a context where this ability was previously denied to them.” [14] Kabeer further conceptualizes empowerment as having three dimensions: material and immaterial resources, which are pre-conditions for agency; agency, which is “the ability to define goals and act upon them”; and achievements, which result from the exercise of agency [14]. Importantly, Kabeer conceptualizes empowerment as a process. Our study aimed to explore, document, and explain that process using qualitative data from women and their husbands in the FAARM trial’s intervention and control households.

## Methods

This study uses qualitative data to examine the influence of an HFP intervention on women’s empowerment in the FAARM trial. FAARM was a cluster-randomized controlled trial conducted in parts of thirteen unions of two rural sub-districts in Habiganj district, Sylhet Division, Bangladesh, from 2015 to 2020 [9]. It evaluated an HFP intervention implemented by HKI jointly with the local non-governmental association Voluntary Association for Rural Development (VARD). The intervention was from mid-2015 to mid-2018, with limited field activities continuing until early 2019 [9]. The primary outcome of the FAARM trial was linear growth among children born to enrolled women. At enrollment, women met eligibility requirements if they had a self-reported age of 30 years or younger, were married, had at least 40 square meters of space for a home garden, and expressed interest to participate in gardening. FAARM enrolled a total of 2706 women in 96 geographically-defined settlements with at least 10 and no more than 65 eligible women, with a minimum distance of 400 m between settlements. Further information on the methods of the FAARM trial can be found in the study protocol paper [9].

In the intervention arm, women’s groups of 8 to 26 members were formed and provided with home gardening equipment and technical training, as well as support for building improved chicken coops. The training topics included home gardening, poultry rearing, hygiene, child care, and nutrition. Groups, with the support of program staff, selected group leaders to assist other group members between trainings and to provide agricultural inputs to their group. Women across all 96 settlements from both the intervention and control arms were monitored over the trial period and assessed for changes in diet and nutritional status. Of the women participating in the FAARM trial, a subset was selected for inclusion in our qualitative evaluation, as described below.

### Participant sampling

Three rounds of qualitative data collection were conducted within FAARM as part of the Gender, Agriculture, and Assets Project, Phase Two (GAAP2) sub-study. While the first two rounds of data collection focused on the social and cultural context of the population, the third round focused on participant experiences during the intervention. The data used in this manuscript come from the third round. To recruit participants for this round of qualitative data collection, settlements where earlier qualitative fieldwork was undertaken were removed from eligibility. Then, up to eight intervention and eight control settlements were randomly selected as study sites, from where data would be collected. Within these areas, in-depth interviews (IDIs) were conducted with two of the randomly selected husband and wife pairs who had completed the pro-WEAI quantitative survey if both husband and wife were available at the time of data collection. From these same areas, data collection staff contacted women enrolled in the trial and their husbands to invite them to participate in focus group discussions (FGDs) until a sample of seven to nine individuals was reached for each group. The original design of the study called for a minimum of 36 IDIs and 12 FGDs of seven to nine participants each. The number of FGDs was based on recommendations to conduct at least two FGDs per stratum, with the four strata in this study being defined by gender and study arm [11]. A total of 44 IDIs were conducted as new themes continued to emerge during data collection, suggesting that saturation had not been reached [15–17]. A total of 93 respondents participated in the 12 FGDs. Table 1 shows the final number of participants by data collection method.

**Table 1:** Number of participants by gender and study arm

	Participants	Intervention (6 settlements)	Control (5 settlements)	Total
In-depth interviews (married couples)	Women	12	10	44 Interviews
	Men	12	10	
Focus group discussions (community members enrolled in FAARM)	Women	3	3	12 Groups
	Men	3	3	

Data were collected in June–July 2019

### Data collection

Data were collected in person in June and July of 2019. The field team consisted of four researchers who were native Bengali speakers, held advanced degrees in anthropology, and were experienced in qualitative interviewing and conducting FGDs in similar settings. The team was comprised of two men and two women so that interviewers and group facilitators would be gender matched to participants.

IDI and FGD guides were developed using components of Kabeer’s conceptual framework of empowerment as well as the FAARM theory of change framework [9, 14]. Topics covered in the guides included self-efficacy, perceived ability to provide the household with nutritious foods, freedom of movement, decision-making processes within the household, financial independence, and nutrition knowledge. The interview and FGD guides were piloted to assess their clarity (including linguistic suitability), local relevance, and the logical flow of questions. Minor changes were made to the guides based on the piloting exercise.

IDIs and FGDs were conducted in Bengali and digitally recorded with participants’ consent. Although most study participants speak the Sylheti dialect of Bengali as their primary language, they are fluent in mainstream Bengali as well. The piloting exercise enabled the research team to identify specific Sylheti terms and phrases that were relevant to the research topics and to clarify their meaning with participants.

While in the field, debriefings were held with the field team at the end of each day. Debriefings were used as an opportunity to practice personal and interpersonal reflexivity, to review and identify issues raised by participants, and to assess saturation [15–18]. Debriefings also led to inductive changes to the guides to further explore issues that were raised by participants. For example, questions were added about the relationships between study participants and mothers-in-law, as well as about food procurement strategies during times when gardens were less productive. Data collection ceased once saturation had been reached.

### Data analysis

**Fig. 1:**
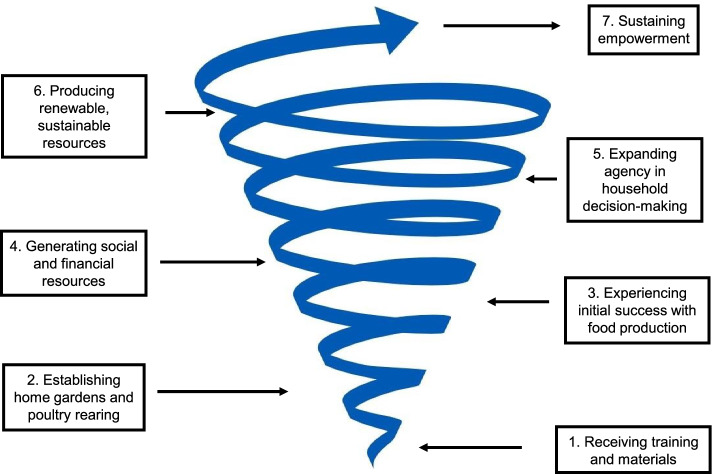
Pathway to empowerment through a homestead food production program

All data were transcribed verbatim from audio recordings in Bengali, translated into English, and deidentified prior to analysis. Verbatim transcription was done by the same research team who conducted the IDIs and FGDs, and the local researcher (MAK) checked transcripts against the audio recordings to confirm accuracy and completeness. Transcripts were then translated into English by a professional translator with previous experience in translating qualitative data. The authors (SD, ASW, JLW, MAK and SSS) performed quality checks by reading all transcripts and following up with the translator and the local research team with any questions. During translation, key words and phrases were retained in brackets in the original language, to capture the emic perspective [15]. All data were uploaded into MAXQDA2020 [19]. Two interview transcripts were removed from the dataset after it was found that the respondents had participated in a home gardening intervention prior to the FAARM intervention, which was an exclusion criterion for this analysis. The excluded transcripts are not represented in Fig. 1.

Code development and coding of data were conducted by the first author (SD), and began with listing deductive codes based on topics in the IDI and FGD guides that reflected Kabeer’s theoretical concepts and the FAARM theory of change. Next, transcripts were carefully reviewed and memoed to note emerging issues, which were captured with inductive codes. Once all codes were developed, data were coded with inductive and deductive codes from the codebook over multiple readings. On later readings, new inductive codes were added to the codebook as additional issues were identified. The codebook was refined and fully developed, including definitions for each code, inclusion and exclusion criteria, and sample quotes from the data that exemplified that code. Lastly, the coding was double checked by the first author (SD) for consistency across the dataset and accurate application of codes per the code definitions.

Once all data were coded, we used a modified grounded theory approach to analyze data, which was best suited to explain how participation in the HFP program influenced women’s empowerment [20]. The primary modification to the grounded theory approach was the consideration of existing constructs related to Kabeer’s framework [14]. First, text segments associated with specific codes were reviewed, and thick descriptions were developed that explored issues in detail. Then, the issues raised in the data were compared by family structure or number of children, to identify patterns and commonalities across and within sub-groups of participants.

Categories were then developed inductively by grouping similar codes together into broader categories. Categories were then linked into distinct phases and a chronology which defined the pathway towards empowerment reflected in the data. The process of conceptualization was facilitated by drawing a series of diagrams to visualize the correlations, relationships, and processes being described by participants. Through these analytic tasks, an inductive framework was developed to explain the pathway towards empowerment among HFP program participants. This analytic process involved a continuous return to both the literature and data, referring to existing theory and checking to assess if the framework and its components were grounded in data. Throughout the process, the researchers engaged in regular discussions about the data, aimed at ensuring that our findings were strongly supported by data while also practicing reflexivity [15]. We used the concept-indicator model to ensure each component of our conceptual framework was grounded in data, by returning to the data to check that each element of the framework and their connections reflected participants’ views [20, 21]. The framework was depicted as an upwards spiral, which best reflected the iterative, non-linear pathway towards empowerment that was described by participants. Once the framework was developed, it was compared to Kabeer’s domains of resources, agency, and achievement, each of which became more visible within each stage of the pathway and fit into the upward spiraling process of empowerment. Thus, the newly developed framework builds on Kabeer’s resources-agency-achievements framework to explain more comprehensively the process of empowerment that was reflected in the data from participants.

### Ethics approval and consent to participate

The data analyzed in this study were collected as part of the FAARM trial (ClinicalTrials.gov: NCT02505711) in its role as a member of the GAAP2 consortium. The study protocol was positively reviewed and approved by the ethics committees of the James P Grant School of Public Health at BRAC University in Bangladesh and Heidelberg University in Germany. All study methods were carried out in accordance with relevant guidelines and regulations. All study participants gave written informed consent prior to participation in FAARM, and additionally provided verbal informed consent for IDIs and FGDs. Consent for publication was obtained from all subjects. This analysis used deidentified data.

## Results

Our modified grounded theory analysis resulted in a framework (Fig. 1) depicting the pathway to empowerment through an HFP program within the FAARM intervention. The framework depicts distinct stages in the process of empowerment. The pathway to empowerment begins with the delivery of training and materials to the participants through educational sessions, then progresses through the establishment of home gardens and poultry rearing, successful food production from gardens and poultry, and the subsequent building of self-efficacy, social capital, and spousal support, leading to improved decision-making power among women. Each of these stages is described below.

### Stage one: receiving training and materials

Stage one involves participants receiving resources in the form of materials, technical training, and nutrition education as part of the HFP intervention in the FAARM trial (Fig. 1). Participants consistently identified the training and materials provided through the intervention as a key component to their later success. The HFP intervention was instrumental in facilitating the process of starting a garden by providing assets such as seeds and fencing, reducing barriers women previously experienced in acquiring agricultural materials, and in receiving training on effective gardening techniques. One female participant highlighted the usefulness of the sack gardening technique taught to help participants preserve their crops during floods, stating:

“*...we didn’t use to grow vegetables like this in the past. Whatever we used to grow, we did that on open soil. But now we fill sacks with soil and grow vegetables on that. It is very useful for the rainy season. We learnt that from you.” - (Female, FGD)*

In contrast, households in the control arm consistently complained that they struggled to sustain productive gardens due to flooding, pests, poor soil quality, or other environmental factors, while no households in the intervention arm shared these complaints.

The acquisition of gardening skills and materials also contributed to an increase in motivation among participants. Participants in the intervention arm frequently noted their own prior ‘laziness’ or lack of interest or motivation to garden, compared to their increased motivation or ‘inspiration’ towards gardening following their participation in FAARM. Both men and women noted that their past efforts at homestead gardening were minimal, and often ended in the gardens producing few or no vegetables. Those who had not gardened in the past discussed making excuses about the challenges and barriers of garden preparation such as having too little space or infertile soil. However, after receiving training through the project, they learned how to overcome these barriers to gardening.

The success in stage one was largely dependent on women gaining permission from their husbands to attend the HFP trainings. Although all participants had previously received their husbands’ consent to attend trainings and expressed interest, some women were not permitted by their husbands to leave their homes when the intervention began. Some women were able to negotiate with their husbands, either by convincing them of the merit of the meetings or by asking their husbands or another family member to escort them. However, some women were not able to successfully navigate the situation. Instead, the husband attended the meetings without the woman, the mother-in-law attended, or nobody attended from that household.

### Stage two: establishing home gardens and poultry rearing

Stage two represents an expression of agency in which participants began to establish their own home gardens and to raise poultry (Fig. 1). Many women reported that their husbands initially were not interested in assisting with the garden. However, after receiving initial training through the project, most women were able describe the potential advantages of gardening, and convince their husbands to support them to begin gardening at home. One participant describes the process of negotiating with her husband, outlining how she started small and eventually reached her goal of a full-sized home garden:

“...*one day suddenly I said to [my husband] … How can we make income for our family? I said to him there is a … project called FAARM, and they will give seeds … I said to him that if you will help me then it will be good for us... At first, I have planted 2-3 plants and then I said to him that there are good vegetables that I get from our plants. Then I said that the way we are growing two plants, in the same way we can grow four plants... That way I have done it.” - (Female, IDI)*

This type of early negotiation is an example of women strategically applying their agency to manifest a situation that they believed to be beneficial to themselves and their families.

This second stage also represents the application of motivation and is represented here by the women who, with or without the support of their spouses, were able to apply the trainings, use materials, and start their gardens. Motivation was especially important to women working on a plot of land that they had previously deemed unsuitable for agriculture due to size, location, or soil quality. These previous barriers to success had discouraged these women from making sincere efforts at gardening in the past, and they had to attempt gardening in new ways and with greater effort than before to overcome them.

### Stage three: experiencing initial success with food production

This stage is marked by the achievement of the first harvest from the home garden, or when chickens had laid enough eggs to sell or consume. As intervention participants began to receive material outputs from their efforts, this led to a further increase in motivation. Many participants reinforced this point, speaking of the confidence, motivation, and self-efficacy earned when they saw their gardens becoming productive. In particular, many participants said that they were confident they could provide nutritious foods to their families even during times of economic hardship. One participant described the change in food availability and the effect it had on her and her children’s diet:

*“We don’t have to buy most of the foods nowadays. We eat those foods [grown in the garden] and serve those to our children. We don’t have to serve a small amount of food to our children thinking of the money we have spent for that food. When we used to buy all those foods, we gave the food to our children at first and ate the remaining after that. But now we grow all those foods and we all can eat the amount of foods we need.”* - *(Female, FGD)*

This stage marked a distinct difference between the intervention and control households regarding the effects of poverty. Where participants from both arms noted poverty as having a negative impact on their lives, control households predominantly discussed food availability as their primary struggle. Conversely, intervention households discussed a more diverse array of topics including access to education, healthcare, or clothing.

This stage was also the point at which previously unsupportive husbands became supportive and, in some cases, began contributing to the garden. This most often was a result of husbands recognizing the benefits of having a successful garden, both for household consumption and monetary profit. A female participant stated:

“*When I plant a particular vegetable and get a good yield, my husband becomes interested in growing vegetables. He starts thinking that he should also help me so that we can get an even better yield together.” - (Female, FGD)*

The husbands’ support proved very important as the fences and chicken coops began to require repairs, and wives relied on their husbands to fix them. If the fences or coops were not fixed, production was likely to become less successful in subsequent harvests.

### Stage four: generating social and financial resources

This stage occurs nearly simultaneously with stage three and represents the acquisition of additional resources through surplus food production. As the gardens and poultry became productive, there were three ways the produce could be used: they could be consumed by the members of the household, they could be sold for profit, and they could be given away to other community members. While all households reported consuming produce from their own gardens, they were also likely to either sell or give away surplus to their neighbors. Selling the surplus produce provided income. Many of the women who chose to sell their surplus produce reported retaining control over the income generated by the sales, and for most this was their only source of purchasing power. This strategy allowed women to spend the income on personal needs, family needs, additional food, their children’s education, or to save it. Women who chose to give surplus produce away to neighbors reported generating resources in the form of social capital. A family who was given produce for free was more likely to give produce back later when they had extra to give or could be asked for other favors. One participant outlined this dynamic as follows:

*“I give [my neighbor] vegetables. They used to ask, ‘how can I grow these vegetables,’ and to please give them some. It is good to eat. ‘How can I grow these?’ they want to know, so I told them how. [FAARM] taught us the way to plant a tree, so I just told them about that, and they did it in the same way. They told me to come and [take some pumpkin] because I taught them how to do it. Pumpkin they grew on their plant, they said I can have it and can eat it” - (Female, IDI)*

Participants in communities who worked together in this way often reported frequently trading or borrowing seeds from each other in a way that supported and benefited everyone who participated.

The ability to sell or gift produce and reap the financial and social resources was a function of how much produce was grown and the size of the household. A household with many individuals was unlikely to have surplus, as was a household with a small garden. Households with extended family living nearby often felt compelled to give extra produce to those family members, which also became a barrier to reaping other benefits from surplus production, as they were neither able to sell that surplus, or use it to generate social resources outside the family.

### Stage five: expanding agency in household decision-making

During this stage, women began to experience increased voice in household decisions as a result of their production of material goods for the family. The demonstration of the ability to make decisions in the garden that resulted in both increased food security and the benefits discussed in stage four led husbands to listen to wives’ opinions more often and with greater respect. Subsequently, women’s input began to become more highly valued in household decisions.

Men noted that as the gardens became more successful, they began to trust their wives’ judgement more, and that their wives were simultaneously ‘braver’ than before about expressing an opinion. Supporting this, the husband of a participant relayed,

“*Women were not aware of many things in the past. They used to be barred from being aware of those things. Now they understand everything. They were restricted to only household chores in the past. But now they are doing different things to support their families … Their husbands, brothers and brothers-in-law take advice from them nowadays.”- (Male, FGD)*

Meanwhile, the women reported increasing self-efficacy and decision-making power within their households as a result of contributing material resources to their families. One woman observed the change as follows:

“*Now [my husband] sees the income and expenditure that is happening in our family. Decisions that I take he sees become fruitful for our family. By this way the change happened.” - (Female, IDI).*

### Stage six: maintaining a renewable supply of resources

Stage six establishes the achievement of long-term sustainability of independence as the participants demonstrated an ability to store seeds and maintain their gardens in the absence of the intervention. By this stage, participants were no longer receiving seeds from FAARM and were applying the techniques they learned for harvesting and storing seeds. Additionally, this stage may also be marked by the chickens continually supplying enough eggs to be sold or consumed and to maintain the current population. The maintenance of gardens and poultry at this stage represents the achievement of habit building and long-term success.

This stage also marked the start of community support around trading seeds. This involved participants with fruitful gardens giving seeds away to those who did not manage to store any, and teaching others in the community effective gardening techniques. This behavior was most evident in communities that favored sharing their surplus produce with neighbors over selling it and reinforced a social norm of community support.

In one settlement, participants unanimously reported sharing seeds and excess produce with those who were lacking, despite the encouragement from the FAARM implementation team to sell seeds and surplus produce. This was done with the general understanding that they would support one another and share surplus produce that was grown, which was beneficial to both parties in the long term. One participant stated,

“*Suppose, I have not stored the seeds and I don’t have seeds now. I can borrow some seeds from her. In the same way she can also borrow some seeds from me.... [The HFP trainer] told us that we can sell the seeds as well. But we don’t do that. We give seeds to each other for free.”- (Female, FGD)*

### Stage seven: sustaining empowerment

This final stage of the pathway marks a sustained improvement in empowerment in the female participants. By the conclusion of the intervention, women who had succeeded in gardening, leveraging surplus, negotiating within their households, and maintaining a renewable supply of resources reported a sustained change in the decision-making dynamics of their households. Both men and women noted a change in the involvement of wives when making larger decisions regarding finances and their children’s education, rather than only the small day-to-day decisions the women were responsible for before, such as what to cook. One husband describes this change over time as a community-wide occurrence, stating:

“*Women’s opinions were not considered seriously four years back. But now their opinions are considered seriously. We see that women’s opinions are taken in every family nowadays. I think, this is a significant change... The women are now participating in some productive and income generating works. Therefore, they have the courage to give their opinions to their husbands.*” - *(Male, FGD)*

## Discussion

This study developed a novel framework to describe a pathway to empowerment among female participants in an HFP intervention, as implemented in the FAARM trial. Our framework builds on Kabeer’s domains of empowerment that include resources, agency, and achievement [14]. Our empowerment framework depicts an iterative process of resource acquisition, agency development, and achievements, and is expressed as an upwards spiral leading towards sustained empowerment. As such, our framework depicts an application of Kabeer’s domains and details the nuances of the pathway to empowerment within this HFP intervention. In this discussion, we expand on the dimensions of agency displayed by participants, along with the importance of negotiation skills, spousal support, and social networks among women.

### Psychological dimensions of agency

Women participating in the HFP intervention described the development of several psychological dimensions of agency, including motivation and self-efficacy, which have been under-studied in this context [22]. In particular, stage two, establishing home gardens, represents the application of motivation when participants returned home to begin their own gardens after attending HFP trainings. Kabeer recognizes motivation as an aspect of “sense of agency”, or the “power within”. She further describes it as the purpose that individuals bring to their activity, with agency defined as “the ability to define one’s goals and act upon them.” [14] It follows that a motivating force to initiate action would be important in overcoming the initial inertia and energy inputs required to begin a home garden.

Motivation, confidence, and sense of control all contribute to one’s self-efficacy and overall sense of agency [22]. HFP intervention participants described increased confidence alongside increased motivation, particularly when describing their ability to provide nutritious foods to their families and to make household decisions that benefit their families. They also described a process of increasing self-efficacy that aligns with existing theory [23]. In particular, performance accomplishment, or mastery over a “difficult or previously feared task,” is considered to be the most effective method through which self-efficacy can be learned or developed [23]. The HFP intervention fostered performance accomplishment by setting participants up for success in a task (gardening and poultry rearing) in which many had not experienced prior success, due to a variety of challenges and perceived barriers. In addition, vicarious experiences, or learning through observing others, is recognized as another effective method for building self-efficacy [23]. HFP intervention participants had the opportunity to witness the achievements of others similar to themselves through their fellow group members and therefore to increase their own self-efficacy through these vicarious experiences.

### Negotiation as an expression of agency

Women in this study also described a process in which an increase in resources led to an increase in bargaining power, which aligns well with the literature. For example, Doss found that acquisition and ownership of assets was linked to improved bargaining power, with asset ownership leading to bargaining power and not the reverse [24]. In the FAARM intervention arm, the initial transfer of resources to women as part of the HFP program led to increased bargaining and negotiation, initially for additional resources or support from their husbands and later for other matters in their homes, based on their status as a resource-generating member of the household. This finding also reflects prior research from South Asia which suggests that bargaining power (or negotiation) related to food allocation is determined by an individual’s utility within their home [25]. By participating in resource-generating activities, the women raise their utility, thereby raising their bargaining power. This improvement in bargaining power can also influence more equal food allocation behaviors within households, along with corresponding improvements in women’s social status within that household [26].

Negotiation is a key component of agency, especially for this population of women in Bangladesh. Kabeer states about South Asian women that “the renegotiation of power relations … is often precisely about changes in informal decision-making, with women opting for private forms of empowerment, which retain intact the public image, and honor of the traditional decision-maker but which nevertheless increases women’s ‘backstage’ influence in decision-making processes.” [14] Negotiation can be considered a form of voice, which has been defined as “the right and ability to enter into the household bargaining process” [27] and “the ability to articulate practical needs and strategic interests” [28]. This voice as “backstage influence” is apparent within our study population: while the husbands remain the head of the household, the wives are able to negotiate for their needs and achieve their goals.

### Spousal support as a key resource towards agency

Our results also reinforce the importance of the support of men as a key resource for women’s empowerment. Since women in Bangladesh live within a cultural context that supports many patriarchal principles [29], the support of men in the community, especially their husband, proved crucial at many points during the intervention. The husbands were in a position of power to either support their wives in reaching their goals, or restrict their ability to work, attend trainings, or collect necessary supplies. For example, some participants failed to effectively initiate their home gardens due to mobility restrictions placed on them by their husbands, meaning they rarely or never attended the trainings. This is consistent with the conceptual framework of empowerment from van Eerdewijk et al., which emphasizes the role of men and boys in the transformation of power relations and in women’s empowerment [13].

Kabeer also recognized the importance of spousal support and that it commonly begins after the initiation of a project, particularly once the men realize that their wives could ease their burden as the primary breadwinner if empowered [30]. Kabeer notes that the products that women produce through these agriculture interventions, while insufficient to lift families out of poverty, ease their reliance on “humiliating” dependency-based relationships [30]. Similarly, women and men in our study noted that husbands often contributed to the gardens only after they began to see it as a productive endeavor. Participants also reported that men were consequently happy to rely less on household income to purchase produce from the market, and instead to produce food at home. An additional benefit for men was the ability to gift surplus produce to their neighbors, procuring respect from others and putting them in an advantageous position to receive other favors.

### Social support networks as a key resource towards agency

In addition to spousal support, the importance of social support and connections with other women are other key components of empowerment identified in our study. The HFP program facilitated connections with other women as it was implemented through a group structure. We found that the participants in the intervention arm who had the most productive gardens and greatest improvements in agency reported that much of their success was attributable to support not only from husbands but also from other women. This reinforces findings from another qualitative study of an HFP intervention in Nepal, which found that participants who had long-term success producing and selling vegetables reported receiving group support from other women [31]. Other research similarly indicates that women’s groups can play an important role in boosting agency among women [8]. While women in the FAARM intervention arm established gardens individually within their homesteads, the HFP training groups may have promoted a sense of collective solidarity. Kabeer notes that “collective solidarity” between women in public spaces is crucial to the development of women’s empowerment and describes women supporting each other in their collective work towards shared interests [14]. This phenomenon was most evident in one settlement, described above, where women shared resources, such as seeds, rather than selling them. Women in this settlement also reported strong and consistent support from their group leader and overall collaboration.

## Conclusions

Our study contributes important evidence on the benefits of a nutrition-sensitive intervention as an approach to sustainably increase women’s empowerment. By conceptualizing the pathway to women’s empowerment taken by participants of a HFP program within the FAARM trial, we were able to identify a series of stages at which participants were eligible for either success and progression, or alternately, pathway interruption. The findings of this study suggest that the women participating in the HFP intervention were successful in their gardens and in increasing their empowerment when they were able to leverage a variety of resources, some of which were provided by the intervention, and some of which were developed from skills learned and fostered through collaboration. The most meaningful improvements in empowerment occurred among participants who were able to produce beyond their household consumption and successfully leverage surplus resources to gain higher utility and therefore bargaining power in their household. Future agriculture-nutrition interventions should seek to mitigate barriers and replicate successes by building self-efficacy via skill development [23], strategically involving men, and creating a system among participants for building their social support networks and social capital, wherein participants meet outside of structured intervention-led sessions. This could be achieved through the inclusion of gender transformative trainings to more explicitly encourage the support of husbands, as well as some technical training for men in the form of garden or chicken coop maintenance, which is seen as a more masculine duty, or in specific nutrition and hygiene education sessions for men.

## Data Availability

The datasets generated and/or analyzed during the current study are not publicly available due to limitations of ethical approval involving confidentiality and anonymity but are available from the corresponding author on reasonable request.

## Abbreviations

FAARM: Food and Agricultural Approaches to Reducing Malnutrition
FGD: Focus group discussion
GAAP2: Gender, Agriculture, and Assets Project, Phase Two
HKI: Helen Keller International
HFP: Homestead food production
IDI: In-depth interview
WEAI: Women’s Empowerment in Agriculture Index
